# The final proteolytic step in transmembrane signaling of multiple RsgI anti-σ factors in *Clostridium thermocellum*

**DOI:** 10.1042/BSR20253055

**Published:** 2025-04-09

**Authors:** Wen Wen, Chao Chen, Qiu Cui, Jinsong Xuan, Yingang Feng

**Affiliations:** 1 Department of Bioscience and Bioengineering, School of Chemistry and Biological Engineering, University of Science and Technology Beijing, Beijing 100083, China; 2 CAS Key Laboratory of Biofuels, Shandong Provincial Key Laboratory of Synthetic Biology, Shandong Engineering Laboratory of Single Cell Oil, Qingdao Institute of Bioenergy and Bioprocess Technology, Chinese Academy of Sciences, Qingdao 266101, China; 3 Qingdao Engineering Laboratory of Single Cell Oil, Qingdao New Energy Shandong Laboratory, Qingdao 266101, China; 4 Shandong Energy Institute, Qingdao 266101, China; 5 University of Chinese Academy of Sciences, Beijing 100049, China

**Keywords:** σ factor, anti-σ factor, AAA+ ATPase, cellulosome, ClpP protease, regulation

## Abstract

In *Clostridium thermocellum*, there are nine RsgI factors responsible for sensing different types of substrates and regulating the transcription and expression of cellulosome genes. Within the signaling pathway of RsgI, the membrane protease RseP cleaves RsgI in its transmembrane helix, thus releasing the N-terminal fragment of RsgI from the membrane. This released RsgI N-terminal fragment is subsequently recognized and degraded by a cytoplasmic protease complex consisting of an AAA+ ATPase and ClpP protease. Previous research showed that the ClpXP complex, comprising ClpX and ClpP, is capable of recognizing and degrading the N-terminal fragment of RsgI6. However, due to the low conservation of the transmembrane helical region of RsgI, it remains unclear whether other RsgIs are similarly recognized and degraded by the same unfoldase. In this study, we employed *in vitro* protease assays to examine the recognition and degradation of the N-terminal fragment of each RsgI by various ClpP-unfoldase complexes. Results confirm that ClpXP is responsible for degrading the N-terminal fragments of all RsgI proteins in *C. thermocellum*, suggesting a degree of sequence promiscuity in substrate recognition by ClpXP. ClpXP can recognize multiple XAA sites in the transmembrane helix region of RsgI. Moreover, we unexpectedly discovered that the cytoplasmic domain influences the degradation of RsgI2-NF by ClpXP in our *in vitro* assay. This study provides new insights into understanding the complex regulatory mechanisms of cellulosome genes and the role of AAA+ proteases in *C. thermocellum*, thereby offering critical clues for unraveling the internal regulatory networks of bacteria.

## Introduction

Bacteria respond to extracellular nutrients, inhibitors, and other stimuli through a variety of mechanisms. One of the most prevalent mechanisms is via alternative σ/anti-σ factor pairs. The σ factor, a component of RNA polymerase, is responsible for recognizing promoters and initiating gene transcription, while the anti-σ factor typically senses signals and activates the σ factor. Bacteria usually encode several different types of σ/anti-σ factors to respond to various stimuli. *Clostridium thermocellum* (also known as *Ruminiclostridium thermocellum*, *Hungateiclostridium thermocellum*, and *Acetivibrio thermocellus*) has a great potential in the field of lignocellulose bioconversion due to its ability to efficiently degrade cellulose [1,2]. This degradation ability originates from the secreted multienzyme complexes, known as cellulosomes, which consist of complex components with modular characteristics [3–5]. The cellulosomal components are transcriptionally regulated according to the presence and types of extracellular substrates [6]. This regulation is primarily achieved through a signaling pathway mediated by multiple pairs of σ^I^ (SigI) and their cognate anti-σ^I^ (RsgI) factors [7,8].

**Figure 1:**
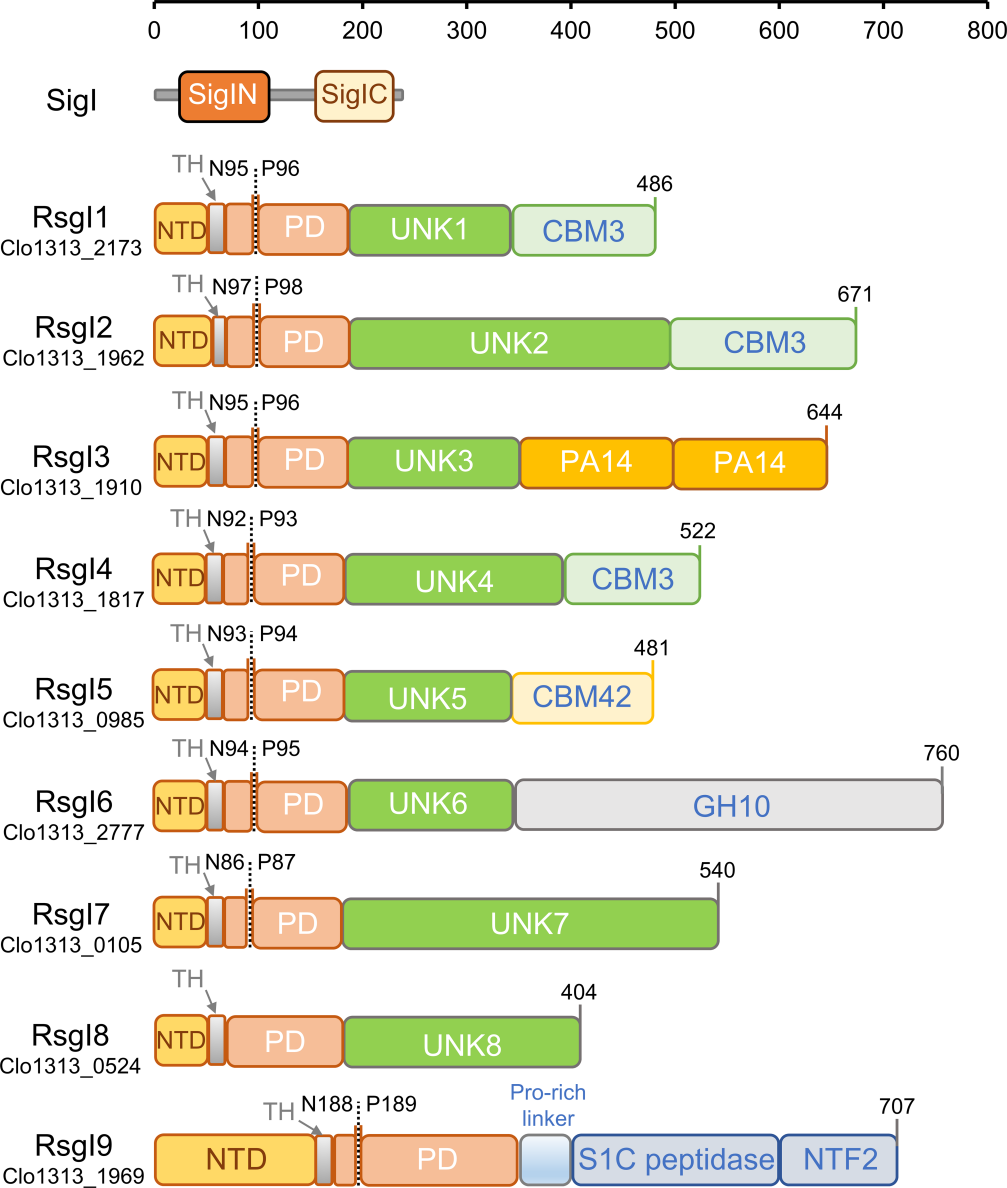
The domain organization of SigI and nine RsgI factors in *C. thermocellum* DSM1313. SigI contains an N-terminal domain (SigIN) and a C-terminal domain (SigIC), responsible for recognizing the promoter -10 and -35 elements, respectively. Each RsgI contains an N-terminal intracellular domain (NTD), a transmembrane helix (TH), a periplasmic domain (PD), and a divergent C-terminal sensor domain which could be a carbohydrate-binding module, a glycosidase hydrolase domain, a peptidase-like domain, or a domain of unknown function (UNK). CBM3, a family 3 CBM that targets cellulose; CBM42, a family 42 CBM that binds to arabinose; PA14, a tandem protective antigen 14 motif that recognizes pectin; GH10, a family 10 glycosidase hydrolase that targets both xylan and cellulose; S1C peptidase, a protease-like domain that is capable of binding cellulose; NTF2, a nuclear transport factor 2-like domain.

**Figure 2:**
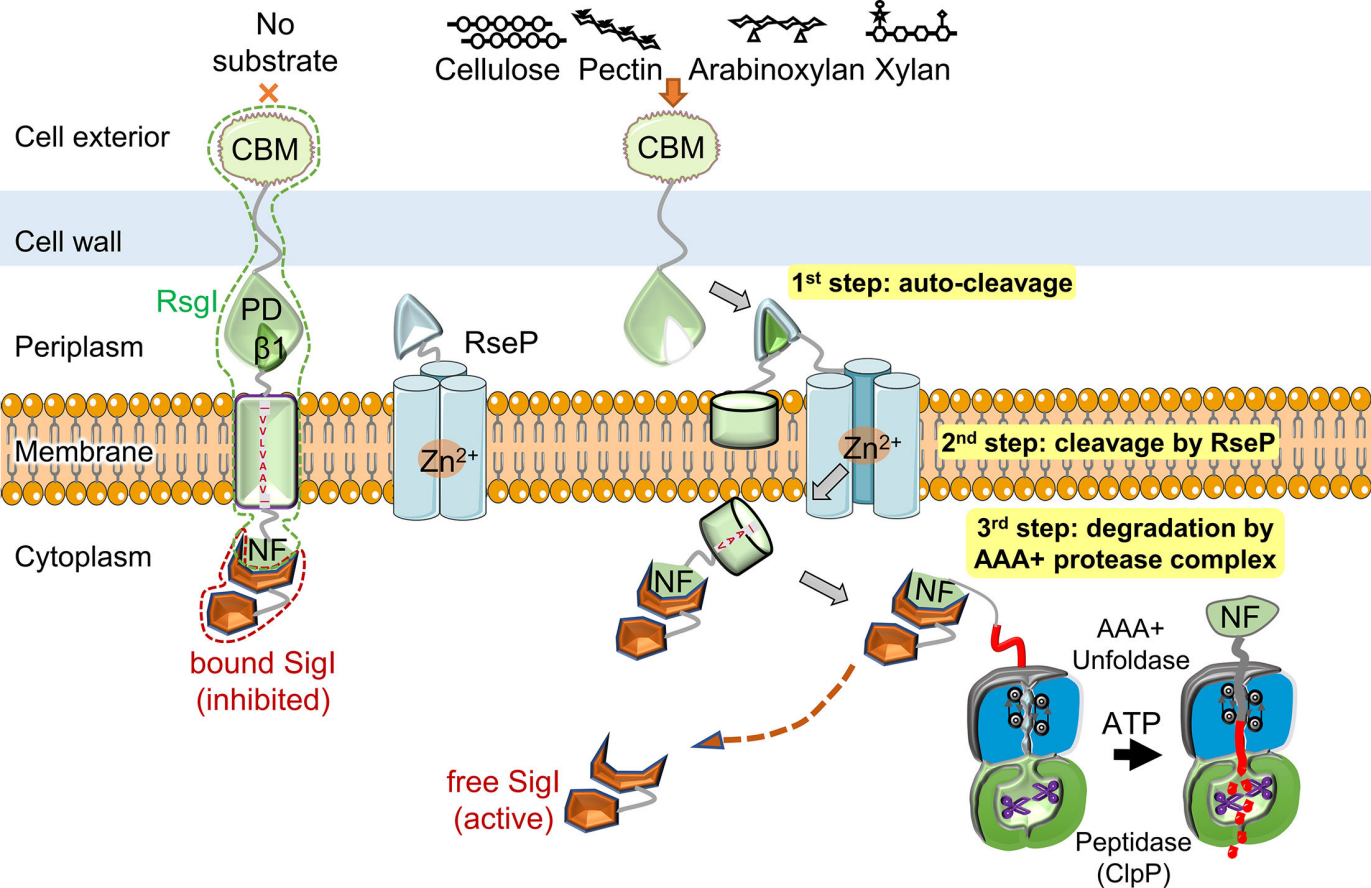
Model of transmembrane signal transduction of RsgI in *C. thermocellum*. The first step of the signal transduction is the autocleavage of the periplasmic domain (PD) of RsgI and the β1 separation from the PD. The second step is the cleavage in the membrane by the protease RseP. The final step (3^rd^ step) is mediated by the AAA+ protease complex which is composed of an AAA+ ATPase as unfoldase for substrate recognition and the peptidase ClpP for substrate degradation. The RsgI and SigI in the inhibited state are indicated by dashed green and red curves, respectively. CBM, Carbohydrate-binding module; PD, periplasmic domain of RsgI; NF, N-terminal fragment of RsgI.

The σ^I^ and anti-σ^I^ factors, prevalent in Bacilli and Clostridia, play pivotal roles in the heat stress response, virulence, and polysaccharide sensing [9,10]. *C. thermocellum* possesses eight pairs of σ^I^/anti-σ^I^ and an individual RsgI9 factor [11–14] (Figure 1). The SigI factor belongs to the σ^70^ family of σ factors with an N-terminal domain and a C-terminal domain that recognize the -10 and -35 elements, respectively, of target promoters. The RsgI protein is a transmembrane protein, consisting of an N-terminal intracellular domain (NTD), a transmembrane helix, a periplasmic domain, a trans-wall region, and an extracellular domain. The C-terminal extracellular domain of RsgI serves as a substrate sensor capable of binding to extracellular carbohydrate substrates in cellulosome regulation. In the absence of substrates, the intracellular N-terminal region of RsgI specifically binds to SigI, consequently inhibiting SigI activity. Upon binding of extracellular substrates to the C-terminal sensor domain of RsgI, a cascade of signal transduction events is triggered, transmitting the signal into the cell and ultimately releasing the RsgI-bound SigI factor. The released SigI factor then recruits RNA polymerase to initiate the transcription and expression of specific cellulosome genes, altering the enzyme composition of the cellulosome (Figure 2). This dynamic regulation of cellulosome components according to the substrate type is one of the key reasons why cellulosome-producing strains can efficiently degrade lignocellulosic substrates [15–19].

In recent years, considerable advances have been made in understanding the regulatory mechanism of cellulosome genes by SigI-RsgI in *C. thermocellum*. Wei et al. revealed that the N-terminal domain of RsgI specifically binds to the C-terminal domain of the cognate SigI, thereby inhibiting SigI activity [9,20]. Chen et al. discovered that the periplasmic domain of RsgI constitutes a novel autocleavage domain, which mimics the first enzymatic cleavage in the regulated intramembrane proteolysis (RIP) signaling pathway [21]. In the classic RIP pathway, three proteases are involved: site-1 protease for the initial cleavage in the extracytoplasmic part of the anti-σ factor, site-2 protease for cleavage in the membrane, and a cytoplasmic protease for final degradation of the anti-σ factor [21]. These three steps form a coordinated proteolytic cascade across three cellular compartments. The unique autocleavage of RsgI in the periplasmic domain resembles the first cleavage event for initiation of signal transduction [10,22]. The membrane-bound protease RseP and the cytoplasmic protease ClpXP have been identified to be responsible for the second and third enzymatic cleavages in the signal transduction of RsgI6, thus elucidating the specific mechanism of RsgI-based transmembrane signaling [22]. Through cryo-electron microscopy structure determination and mutational analysis of the transcription open complex formed by SigI1 or SigI6 with RNA polymerase and the *sigI* gene promoter, Li et al. elucidated how SigI binds to the RNAP core enzyme in the active state and specifically recognizes the corresponding promoter for cellulosome regulation [23]. They also discovered that SigI represents a new group (Group V) within the Sigma70 family [23–26].

It should be noted that the transmembrane signaling mechanism of RsgI in *C. thermocellum* has only been elucidated through research on RsgI6 in the aforementioned study, while the mechanism for the other 8 RsgIs remains to be experimentally confirmed [22,27]. Although the majority of different RsgIs’ transmembrane helices contain a C-terminal XAA motif, similar to SsrA-tagged proteins as a substrate for ClpXP, the sequence conservation in other regions is notably low. This raises the question of whether all these RsgIs can be degraded by ClpXP [21,28–32]. ClpXP is an AAA+ protease complex formed by the ClpP protease core and the AAA+ ATPase ClpX [33,34], where ClpX is an unfoldase responsible for substrate recognition [35–38]. Besides ClpX, *C. thermocellum* also encodes other AAA+ ATPases such as ClpA, ClpC, and ClpE [35]. Some AAA+ ATPases require adaptor proteins, which are essential for enhancing specificity or polymer assembly [39]. For example, MecA is necessary for the stable assembly of active ClpC hexamers in many strains and has been shown to facilitate ClpCP in degrading the transcription factor SpoIIAB [40–42]. Another example is the adaptor protein ClpS, which lacks ATPase activity and binds to the N-terminal domain of ClpA [43–47]. The low sequence conservation of the C-terminal regions of different RsgIs and the presence of multiple AAA+ proteases in *C. thermocellum* inspired us to investigate the third proteolytic step of each RsgI in the signal transduction.

In this study, we further analyzed the transmembrane helical regions of nine RsgIs in *C. thermocellum*. Utilizing an *in vitro* ClpP protease–ATP regeneration system, we investigated the degradation of various N-terminal fragments of RsgI (RsgI-NFs) by different types of ClpP protease complexes. This analysis provided insights into the specific mechanism of intracellular proteolysis in the third step of signal transduction for different RsgIs in *C. thermocellum*.

## Materials and methods

### Bacterial strains, growth media, and culture conditions

*C. thermocellum* DSM 1313 was obtained from the DSMZ (German Collection of Microorganisms and Cell Cultures, Braunschweig, Germany). *Escherichia coli* strain Top10 (Sangon Biotech, Shanghai) was used for plasmid constructions, and strain BL21(DE3) or Rosetta (DE3) (Shanghai Sango Biotech) was used for protein overexpression. *E. coli* was cultured in a lysogeny broth (LB, also known as Luria-Bertani) medium. When required, kanamycin (Kan, 50 μg/ml) or chloramphenicol (Cam, 30 μg/ml) was added to the medium. The strains used in this study are listed in Supplementary Table S1.

### DNA manipulation techniques and construction of plasmids

Standard procedures were employed for DNA manipulations, including genomic DNA preparation, PCR, cloning, ligation, and transformation. DNA fragments encoding either N-terminal fragments of RsgIs or full-length Clp proteases were amplified from the genomic DNA of *C. thermocellum* DSM 1313 via PCR, utilizing the appropriate primers as listed in Supplementary Table S2.

The purified PCR products were ligated into the pET28a or the pET28a-SMT3 vector between the *Nde*I/*Bam*HI and *Xho*I digestion sites by standard molecular cloning techniques using restriction enzymes and ligase, or by the ligase-independent cloning technique based on a seamless cloning kit (Vazyme Biotech, Nanjing, China). The resulting plasmids designed for recombinant protein overexpression are listed in Supplementary Table S3. The recombinant proteins ClpX, ClpE, ClpC, ClpP, ClpA, and the adaptor protein factor (MecA) encoded in the pET28a-derived plasmids contain an N-terminal or a C-terminal His_6_-tag. The recombinant proteins encoded in the pET28a-SMT3-derived plasmids, including RsgI N-terminal fragments (RsgI-NFs) and SpoIIAB, contain an N-terminal His_6_-SMT3 tag, which can be removed by the ubiquitin-like-specific protease (ULP1) treatment when necessary.

### Recombinant protein expression and purification

The recombinant plasmids that encoded RsgI-NFs, SpoIIAB, and MecA domains were transformed into *E. coli* Rosetta (DE3) for protein expression. The other plasmids were transformed into *E. coli* BL21 (DE3).

The bacterial cells were cultured overnight in 50 ml of LB medium at 37°C, supplemented with kanamycin (50 μg/ml) and chloramphenicol (30 μg/ml), and then diluted into 1 l of LB medium. When the absorbance at 600 nm reached 0.8, isopropyl-β-D-thiogalactopyranoside was added at a final concentration of 0.5 mM to induce the expression of the target protein for 4–6 h at 37°C. Proteins were induced for expression at 37°C, except for RsgI5-NF-SVA, RsgI7-NF-VAA, ClpA, and RsgI9-NF-IAA, which were induced at 16°C for 12–16 h. The cells were collected by centrifugation at 6000 ***g*** at 25°C for 15 min. The cells were resuspended in a 20-ml binding buffer (20 mM Tris-HCl, 500 mM NaCl, 10 mM imidazole, pH 8.0) and frozen at −80°C. The cells were then thawed at 4°C and lysed by sonication. After centrifugation at 6000 ***g*** for 30 min, the supernatant was applied on a Histrap column and the proteins were eluted with the elution buffer containing 20 mM Tris-HCL, 500 mM NaCl, and 300 mM imidazole at pH 8.0. The eluate was concentrated to 2 ml using Amicon Ultra-15 centrifugal filter units (3 kDa cut-off) (Millipore). The protein solutions were exchanged into the PZ buffer (25 mM HEPES, 200 mM KCl, 5 mM MgCl_2_, 1 mM dithiothreitol, 10% glycerol, pH 7.6) by using a PD-10 desalting column (GE Healthcare, Uppsala, Sweden) and then frozen at −80°C. The purity of the protein was evaluated by sodium dodecyl sulfate–polyacrylamide gel electrophoresis (SDS-PAGE). Unedited images of the SDS-PAGE gels are shown in the Supplementary Materials.

### Western blot

After SDS-PAGE, proteins on the gel were transferred to the nitrocellulose (NC) blotting membrane in an electric field. These membranes were blocked using TBST-blocking buffer (50 mM Tris, 150 mM NaCl, 0.07% Tween-20, 5% skim milk, pH 7.4) for a duration of 2 h. Then, the membranes were incubated with mouse anti-His tag monoclonal antibody (Bioss Antibodies, Beijing, China) overnight at 4°C. Next, the membranes were incubated with a horseradish peroxidase-conjugated goat anti-mouse IgG secondary antibody (Bioss Antibodies, Beijing, China) for 1.5 h. After incubation, the membrane was washed with TBST buffer (50 mM Tris, 150 mM NaCl, 0.07% Tween-20, pH 7.4) three times, each wash lasting 5 min. Finally, the NC membranes were developed using an ECL hypersensitive chemiluminescence reagent (MDBio, Inc., Qingdao, China) and subsequently exposed in a chemiluminescence imager. Unedited images of the Western blot results are provided in the Supplementary Materials.

### Protease assay

The degradation of RsgI-NFs by candidate ClpP proteases was assessed via *in vitro* protease assays according to the previous literature with slight modifications [22]. Briefly, recombinant proteins, including ClpP (3 μM), one of its ATPases (ClpX, ClpE, ClpC, or ClpA) (3 μM), and the adaptor protein factor (3 μM) when necessary, were pre-incubated with an ATP regeneration mix (4 mM ATP, 16 mM creatine phosphate, 20 U/ml creatine kinase) (Shanghai Yuanye Bio-Technology Co. Ltd.) for 15 min at 30°C in the PZ buffer. The substrate (purified RsgI-NF) was then added to the buffer and reacted at 50°C for 40 min. SDS-PAGE was used to detect the degradation of RsgI-NFs. Each assay is repeated at least three times.

## Results

### Analysis of the transmembrane region of RsgIs and ClpP unfoldases in *C. thermocellum*

**Figure 3:**
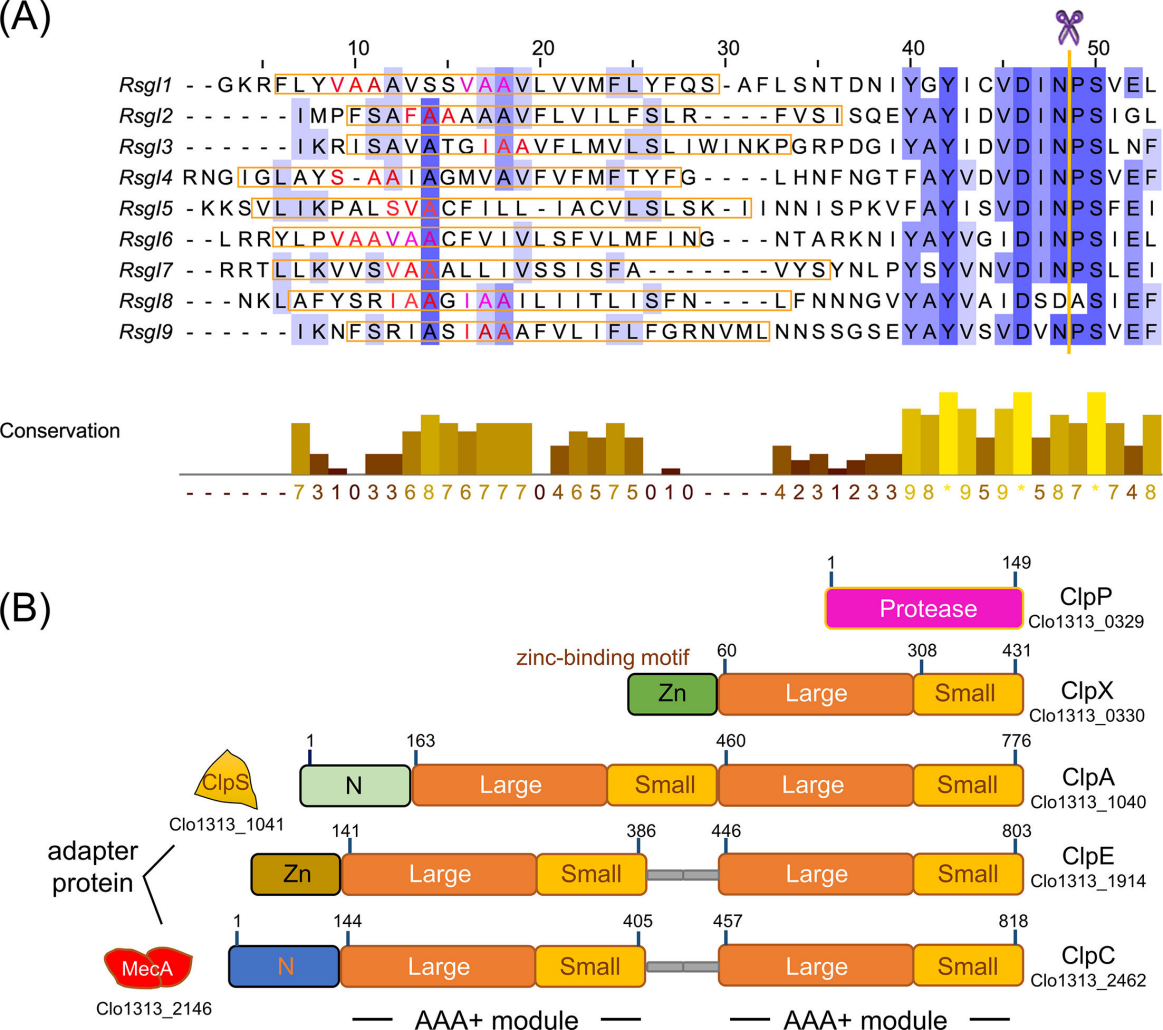
The transmembrane region of nine RsgI factors and the domain organization of four unfoldases in *C. thermocellum* DSM1313. (**A**) Sequence alignment of the transmembrane helix regions of the RsgIs in *C. thermocellum* DSM1313. The transmembrane helices predicted by the TMHMM program are indicated by orange rectangles, and the XAA motifs are shown in red. (**B**) Domain organization and gene locus of potential AAA+ ATPases, ClpP protease, and adaptor proteins in *C. thermocellum* DSM1313.

The transmembrane helices of the nine RsgIs in *C. thermocellum* predicted by the TMHMM program are 23–25 residues in length (Figure 3(A)). These predicted transmembrane sequences mostly contain one or two XAA motifs (with X typically being a hydrophobic amino acid residue), which is reminiscent of the sequence of the SsrA tag recognized by ClpXP [48–51]. RsgI1, RsgI6, and RsgI8 each contain two XAA sequences; RsgI5 does not contain an XAA sequence, with SVA speculated to perform a similar function; RsgI4 has a hydrophilic serine (SAA) at the X position; RsgI1, RsgI2, RsgI7, and RsgI9 have additional alanine residues following the XAA sequence [52]. However, it is important to note that the conservation of sequences near XAA is low, and it remains uncertain whether these sequences can be recognized by ClpXP or other unfoldases of ClpP proteases [53,54]. Four types of Clp unfoldases are present intracellularly in *C. thermocellum* (Figure 3(B)). Each Clp unfoldase comprises one or two AAA+ modules, with each module consisting of a large domain, a small domain, and additional family-specific domains. These Clp unfoldases function as AAA+ ATPase subunits alongside ClpP proteases to exert proteolytic activity. *C. thermocellum* also contains the known adaptor proteins MecA and ClpS. We attempted to express and purify these protein subunits in *E. coli* and successfully obtained soluble expression of most RsgI-NFs containing the NTD and part of the transmembrane helix, ClpP, and most unfoldases. The detailed results of these protein expressions are described in subsequent sections.

### Optimization of the expression of RsgI-NFs in *E. coli*

**Figure 4:**
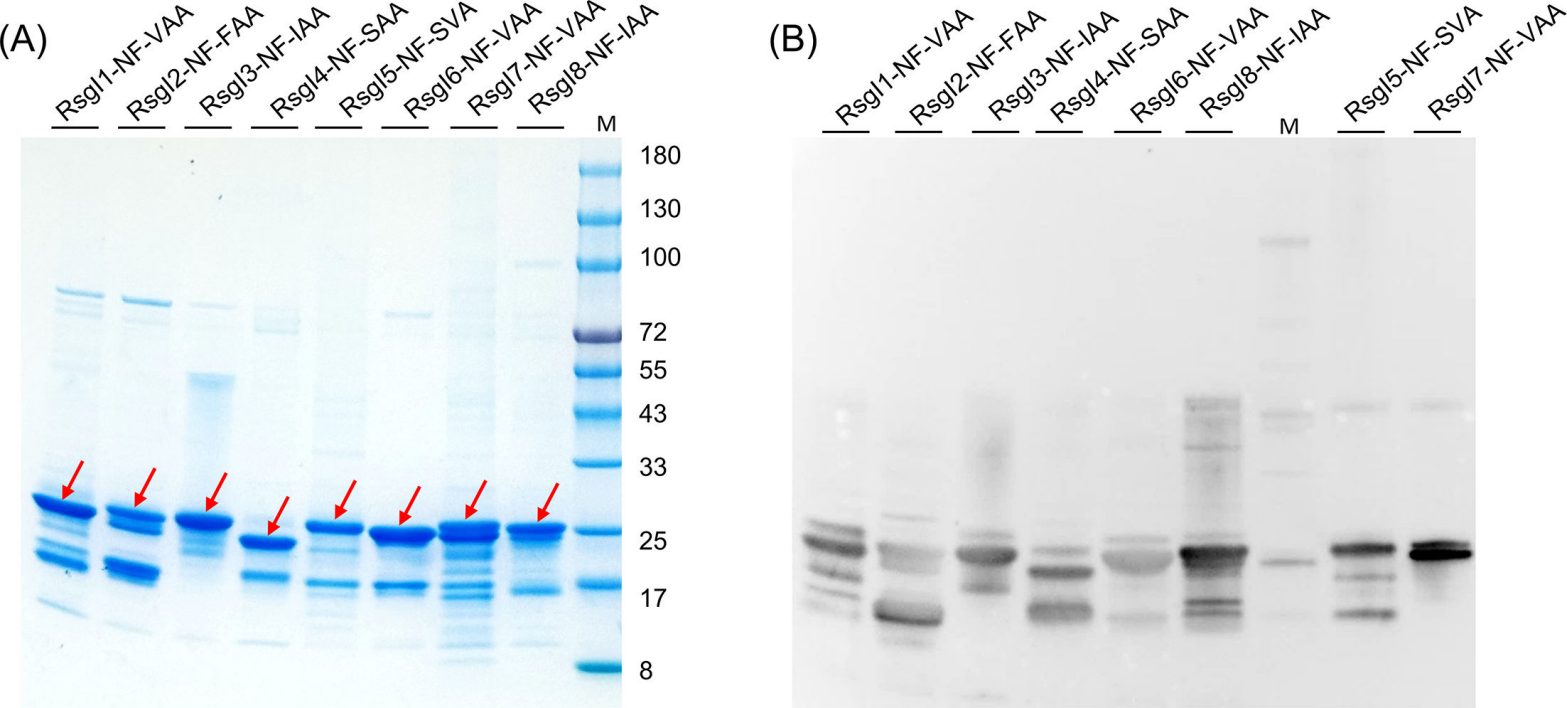
Purified RsgI-NFs. **(A**) SDS-PAGE of the eight purified RsgI-NFs samples. The expected RsgI-NF bands are indicated by red arrows. (**B**) Western blot analysis of the eight purified RsgI-NFs using the anti-His6-tag antibodies.

In previous studies, we demonstrated that RsgI6NFs (VAA1 and VAA2) expressed in *E. coli* can be degraded by ClpXP. However, the small molecular weight of RsgI-NFs (~8 kDa) causes them to migrate to the lower edge of the gel during SDS-PAGE, making their observation difficult. Furthermore, when we attempted to express other RsgI-NFs using the same approach, we encountered issues including poor solubility and protein instability. Consequently, we modified our approach and used plasmids providing an extra His_6_-SMT3 tag fused to the N-terminus of the target proteins. The target proteins were purified using a Ni^2+^ column and then used in the protease assays instead of using cell lysates as previously done. With this expression system, we successfully obtained soluble proteins for the N-terminal fragments of RsgI1 through RsgI8 (Figure 4(A)). It should be noted that all the proteins probably exhibit varying degrees of degradation, resulting in multiple bands below 25 kDa. Specifically, RsgI5-NF-SVA and RsgI7-NF-VAA experienced the most significant degradation, with only very faint bands observable at the expected positions. We then examined the samples eluted at different times from the Ni^2+^ column and found that those eluted later had a lower degree of degradation and showed clear bands at the expected positions for RsgI5-NF-SVA and RsgI7-NF-VAA (Supplementary Figure S1). These fractions were subsequently used for further analysis.

We attempted to express the RsgI9-NF-IAA protein but found its presence in inclusion bodies under both 37°C and 16°C conditions (Supplementary Figure S2). Introducing an SMT3 tag at the N-terminus of RsgI9-NF-IAA did not alleviate this issue. Therefore, further study is needed to address the purification challenges related to RsgIN9.

We conducted a Western blot analysis of the eight purified RsgI-NFs using the anti-His6-tag antibodies (Figure 4(B)). We found that the majority of bands below the anticipated molecular weight (~25 kDa) was detectable via Western blot. This suggests that these bands represent degraded target proteins containing an N-terminal His_6_-tag, which were likely generated before purification.

### Only ClpXP can recognize and degrade most of RsgI-NFs

Our previous study revealed that ClpXP could degrade RsgI6-NF-VAA1 and RsgI6-NF-VAA2, whereas ClpCP and ClpEP were unable to degrade them. ClpC may require the adaptor protein MecA as indicated by literature [39,40], so we incorporated MecA into the ClpCP experiments in this study. Additionally, we found that ClpA unfoldase is also present in *C. thermocellum*, so we conducted assays using ClpAP in this study. While ClpS is an adaptor protein for ClpAP, it inhibits the degradation of SsrA-tagged substrates and enhances the degradation of N-end-rule proteins containing an aromatic or hydrophobic N-terminal residue [43–45]. Since RsgI-NFs are not N-end-rule type of substrates, we did not include ClpS in the ClpAP protease assays for RsgI-NFs.

**Figure 5:**
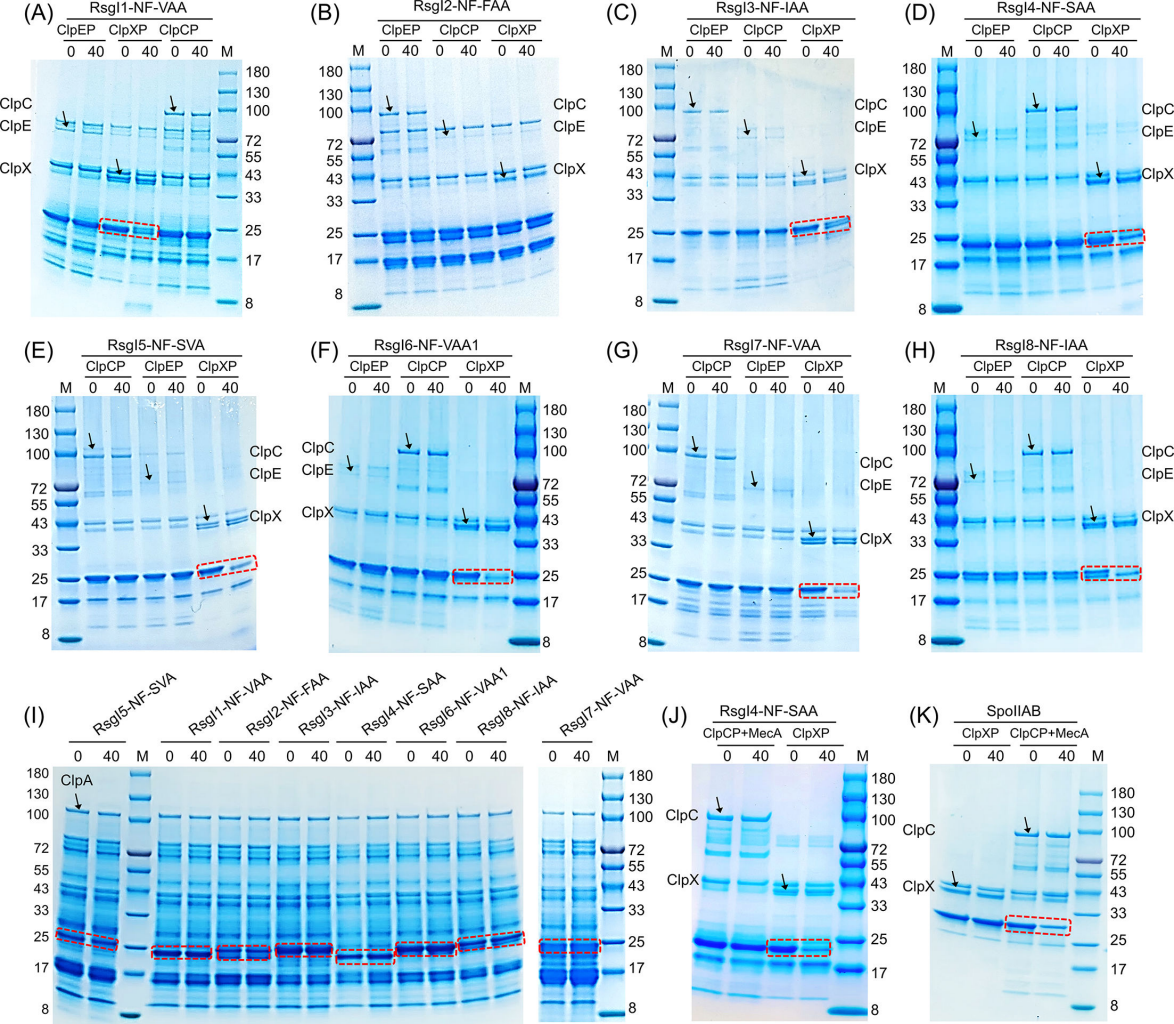
ClpEP, ClpCP, ClpXP, and ClpAP protease assays for RsgI-NFs. 0: before reaction, 40: after 40 min of reaction at 50°C. The bands of RsgI-NFs are indicated by red dashed rectangles. The bands of unfoldases are indicated by black arrows. (**A–H**) ClpEP, ClpCP, and ClpXP protease assays for the RsgI-NF proteins; (**I**) ClpAP protease assay for the RsgI-NF proteins; (**J**) ClpCP with MecA and ClpXP protease assays for the RsgI4-NF-SAA protein; (**K**) ClpCP protease assay for the SpoIIAB (anti-σ^F^) protein.

**Figure 6:**
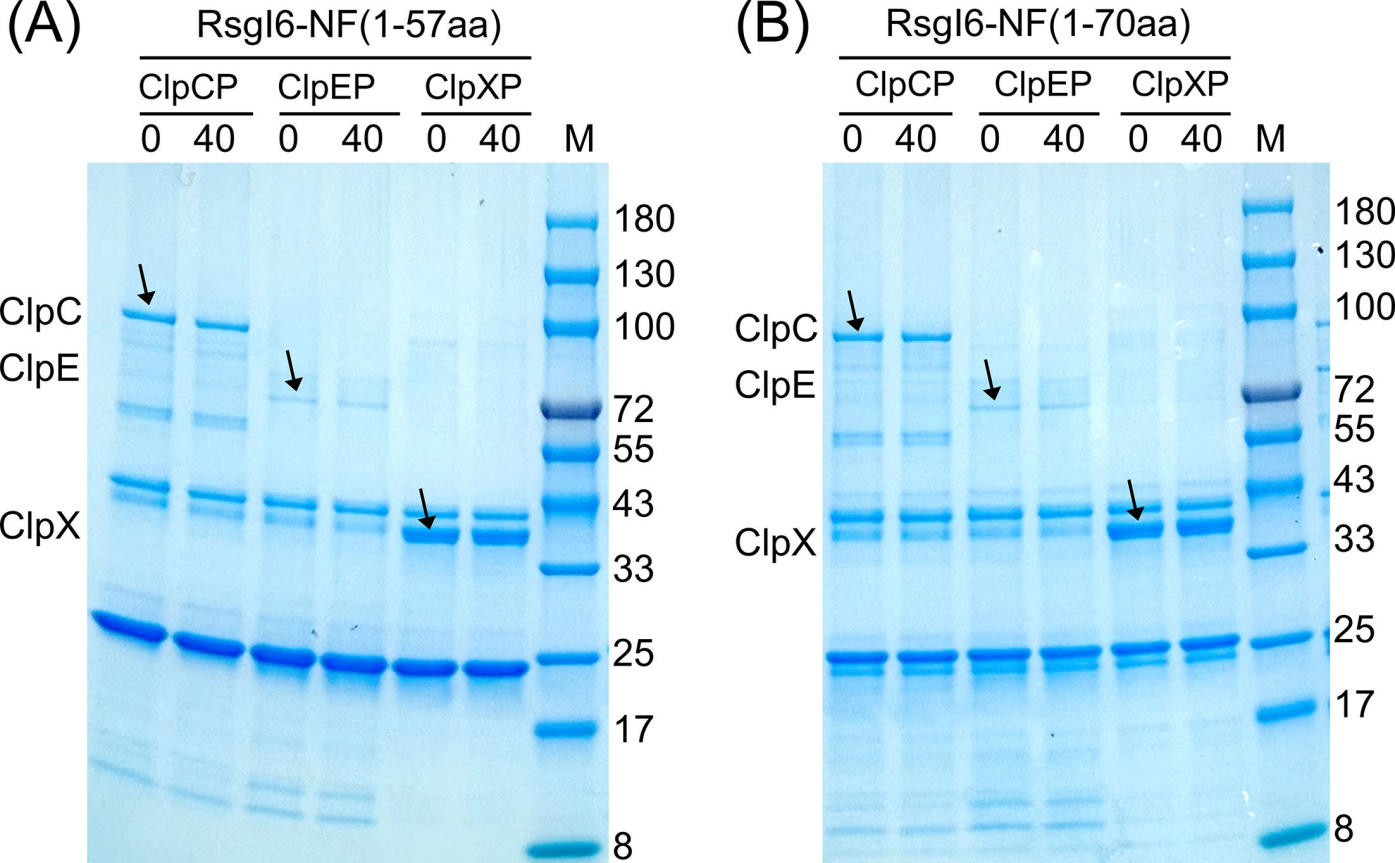
ClpEP, ClpCP, and ClpXP protease assays for RsgI6-NF(1–57aa) and RsgI6-NF(1–70aa). The bands of unfoldases are indicated by black arrows. 0: before reaction, 40: after 40 min of reaction at 50°C.

*In vitro* protease assays using ClpXP, ClpEP, ClpCP, and ClpAP indicated that RsgI1-NF-VAA, RsgI3-NF-IAA, RsgI4-NF-SAA, RsgI5-NF-SVA, RsgI6-NF-VAA1, RsgI7-NF-VAA, and RsgI8-NF-IAA could be degraded by the ClpXP system but not by ClpEP, ClpCP, or ClpAP (Figure 5(A–I)). Unexpectedly, RsgI2-NF-FAA was resistant to degradation by all the ClpP proteases tested. The results for RsgI6-NF-VAA1 using the His_6_-SMT3 tag fused proteins align with the previous study using the protein without the SMT3 tag [22], indicating that the His_6_-SMT3 tag does not influence proteolysis. Two negative control proteins, RsgI6-NF(1–57 aa) and RsgI6-NF(1–70 aa), which contain the NTD only and the NTD plus the whole transmembrane helix, respectively, were used to confirm the significance of the C-terminal XAA motif in the recognition and degradation. Protease assays confirmed that neither of these proteins could be degraded by the AAA+ protease systems (Figure 6).

To further demonstrate the enzymatic activity of the unfoldases we obtained, we chose SpoIIAB, a known ClpC substrate [55] for assessment. By comparing the experimental results using RsgI4-NF and SpoIIAB as substrates, we found that RsgI4-NF is degraded by ClpXP, but not by ClpCP (Figure 5(J)). Conversely, SpoIIAB was found to be degraded by ClpCP and not by ClpXP (Figure 5(K)). This indicated that the ClpCP used in this experiment is enzymatically active, and ClpXP and ClpCP exhibit specificity in recognizing different substrates.

### The recognition of multiple and extended XAA sites in RsgI-NFs by ClpXP

**Figure 7:**
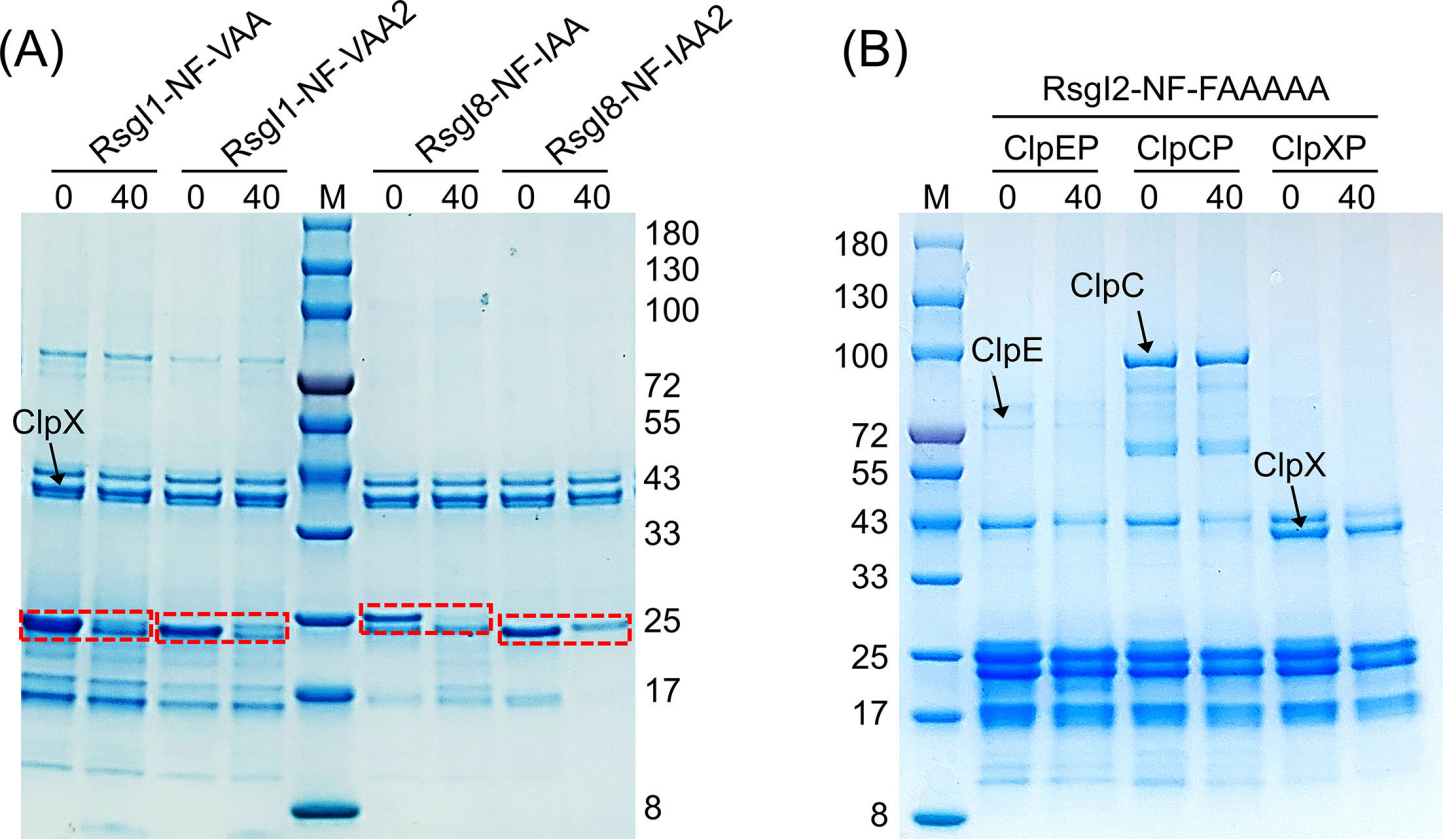
The recognition of multiple and extended XAA sites in RsgI-NFs by ClpXP. The bands of unfoldases are indicated by black arrows. (**A**) ClpX protease assays of RsgI1-NFs and RsgI8-NFs truncated at different XAA motifs in the transmembrane region. (**B**) Protease assay of RsgI2-NF-FAAAAA.

RsgI6 contains two XAA motifs in the transmembrane helix, and our previous study demonstrated that both RsgI6-NFs truncated at each XAA motif can be recognized and degraded by ClpXP. In the present research, we further explored other RsgI factors that contain multiple XAA sequences, as well as those with additional alanine residues following XAA. For both RsgI1 and RsgI8, which contain two XAA sequences, we obtained four soluble target proteins: RsgI1-NF-VAA, RsgI1-NF-VAA2, RsgI8-NF-IAA, and RsgI8-NF-IAA2. Protease assays revealed that all these proteins could be degraded by the ClpXP system, but not by other protease systems (Figure 7(A)).

Notably, no degradation was observed for the RsgI2-NF-FAA protein during the protease assay. RsgI2 possesses three additional alanine residues following the FAA sequence, which led us to hypothesize that a longer C-terminal tail might be necessary for recognition and degradation. Therefore, we constructed three new RsgI2-NFs: RsgI2-NF-FAAA, RsgI2-NF-FAAAA (Supplementary Figure S3), and RsgI2-NF-FAAAAA. However, upon testing these three substrates in the ClpP protease system, none of them could be degraded by ClpXP or other ClpP proteases (Figure 7(B)). Thus, it remains unclear which protease is responsible for recognizing and degrading RsgI2, necessitating further research on this matter.

### ClpXP can recognize the XAA motifs of the transmembrane helices of RsgI2 and RsgI9

To address the problem of non-degradation of RsgI2-NF, we proposed that the intracellular N-terminal domain of RsgI2 could potentially inhibit the degradation. Therefore, we constructed a chimera, RsgI6N-RsgI2TM-NF, which incorporates the N-terminal domain (residues 1–54) of RsgI6 and the N-terminal region (residues 63–68) of the transmembrane helix of RsgI2. This construct was subsequently expressed in *E. coli* via recombinant technique. The same strategy was employed to address the challenges associated with the expression and purification of RsgI9-NF. We constructed a chimera, RsgI6N-RsgI9TM-NF, comprising the N-terminal region (residues 150–158) of the transmembrane helix of RsgI9 and the N-terminal domain of RsgI6. This construct was successfully expressed and purified.

**Figure 8:**
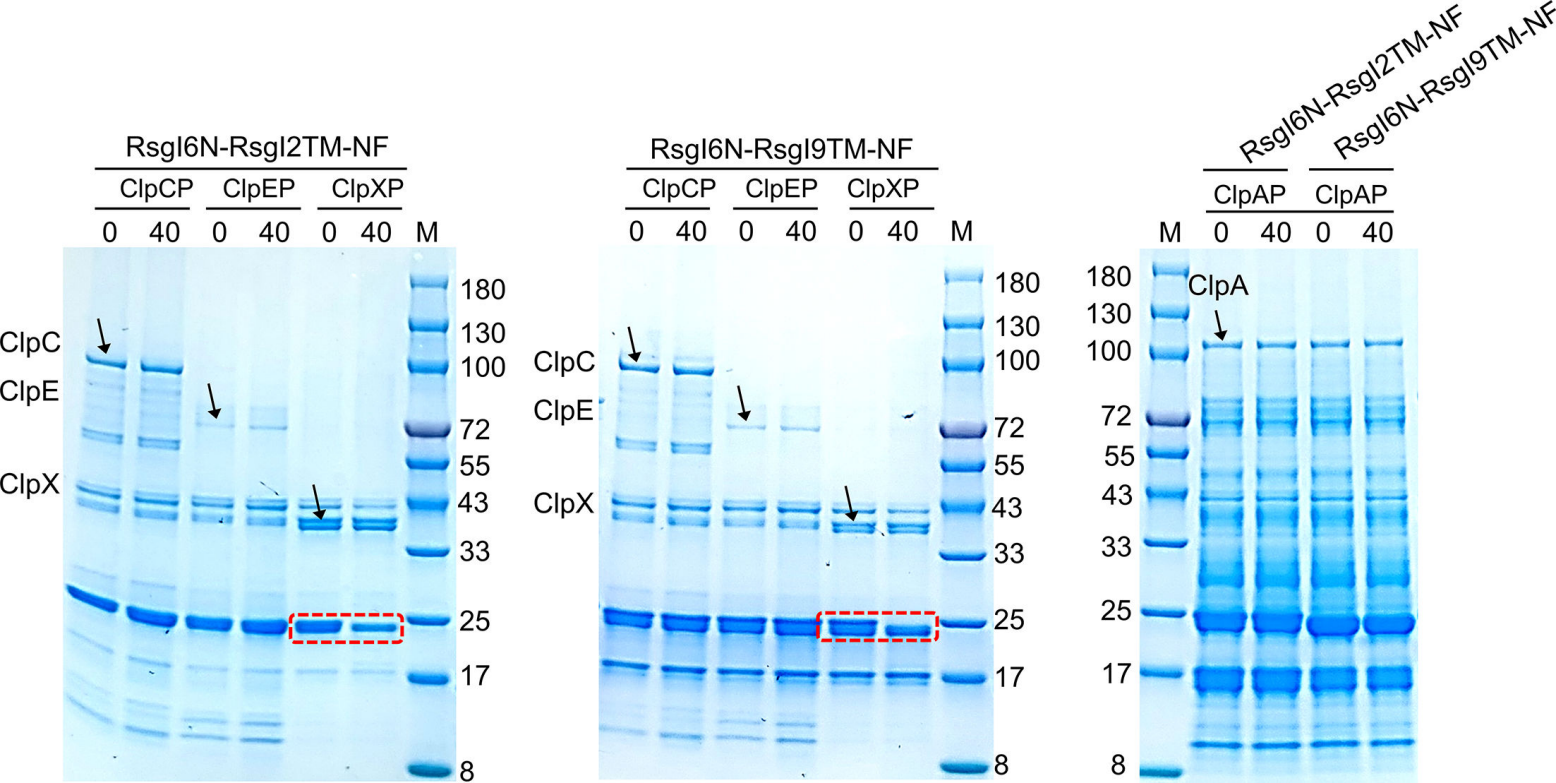
ClpEP, ClpCP, ClpXP, and ClpAP protease assays for RsgI6NTD-RsgI2TM-NF and RsgI6NTD-RsgI9TM-NF. The bands of unfoldases are indicated by black arrows. 0: before reaction, 40: after 40 min of reaction at 50°C.

Using this fusion approach, we successfully obtained the RsgI6NTD-RsgI2TM (63-68)-NF and RsgI6NTD-RsgI9TM (150-158)-NF proteins. Subsequent protease assays revealed that both of these proteins are degradable by the ClpXP system, but not by the ClpEP or ClpCP system. This demonstrates that the XAA motifs in the transmembrane helices of RsgI2 and RsgI9 can be recognized by ClpXP (Figure 8). These findings imply that ClpXP is responsible for the final proteolytic step of RsgI2 and RsgI9. However, further research is needed to investigate the inhibitory mechanism of the intracellular N-terminal domain of RsgI2 on ClpXP recognition.

## Discussion

In this study, we employed the *in vitro* protease assays to explore the final proteolytic step of signal transduction involving multiple RsgIs in *C. thermocellum*. Our findings reveal that the final proteolysis of all nine RsgIs is facilitated by ClpXP. Specifically, RsgI1, RsgI3, RsgI4, RsgI5, RsgI6, RsgI7, and RsgI8 were demonstrated to possess N-terminal fragments, generated at their XAA motifs, which are susceptible to degradation by ClpXP. Additionally, RsgI1, RsgI6, and RsgI8 each contain two XAA motifs within their transmembrane helix, and the N-terminal fragments at either XAA motif can be recognized by ClpXP. While we did not directly observe the degradation of RsgI2-NF and RsgI9-NF by ClpXP, we confirmed that the XAA motifs of their transmembrane helices can be recognized by ClpXP using a chimera with RsgI6. Despite the low sequence conservation of the transmembrane helices of RsgIs, our results indicate that they can be effectively recognized and degraded by ClpXP. This implies that ClpXP primarily relies on a few amino acids at the C-terminus for substrate recognition and plays a pivotal role in RsgI signal transduction in *C. thermocellum*.

It is important to note that variations may exist in the transmembrane signaling mechanisms among the different RsgIs. For instance, although RsgI2 contains an XAA motif, our experiments did not identify its degradation by ClpXP or other proteases. The C-terminal region of RsgI2 serves as a CBM3-type sensor domain capable of recognizing cellulose [56]. Previous transcriptomic data showed a nearly five-fold up-regulation of SigI2 during the later stages of cellulose fermentation [57], indicating the importance of RsgI2. In contrast with other RsgIs, the transmembrane sequences of RsgI2 contain five consecutive alanine residues. Our results demonstrated that RsgI2-NF with truncation at any position within these alanine residues cannot be degraded by ClpP proteases. However, the chimera RsgI6N-RsgI2TM-NF can be recognized by ClpXP. Therefore, the cytoplasmic domain of RsgI2 may also regulate the final proteolysis step of RsgI2. Further studies are needed to elucidate the unexpected regulation mechanism of RsgI2 signaling.

Although our experiments indicated that RsgI8 is degraded by ClpXP, it does not possess a self-cleavage motif in its periplasmic domain and our previous studies have shown that RsgI8 does not undergo self-cleavage in *C. thermocellum*, while the self-cleavage in the periplasmic domain of other RsgIs has been proposed to be essential for initiation of the signaling [22]. Therefore, the mechanism responsible for generating the initial signal for RsgI8 remains unidentified. Studies on the sole RsgI in *Bacillus subtilis*, BsRsgI, have suggested that closure of the self-cleavage site does not completely block its signal transduction, indicating the possible existence of an alternative mechanism for signal generation and transduction [10]. RsgI8 in *C. thermocellum* lacks a distinct extracellular carbohydrate-binding domain, suggesting it might function similarly to BsRsgI, possibly implying other signal sensing capabilities, which need further investigation in the future.

For the ‘orphan’ RsgI factor, RsgI9, in *C. thermocellum*, we were unable to obtain soluble expression and purification of its N-terminal fragment. However, through the use of the chimera RsgI6N-RsgI9TM-NF, we demonstrated that the XAA motif of RsgI9 is recognizable by ClpXP. While the C-terminal sequence of RsgI9 contains a domain homologous to proteases, previous studies have indicated that it possesses polysaccharide-binding ability [13]. The periplasmic domain of RsgI9 exhibits autocleavage capability [58], indicating that RsgI9 may retain its role as a substrate-sensing factor. Identifying the downstream SigI factor for RsgI9 remains a task for further exploration. Sequence analysis reveals that the intracellular region of RsgI9 has a longer sequence than that of the other eight RsgIs. This region contains a β-barrel-like domain similar to that found in other RsgIs and an additional sequence of approximately 100 amino acids between this domain and the transmembrane helix. Both sequence analysis and AlphaFold2 structure prediction suggest that this additional sequence is an intrinsically disordered region. The impact of this region on the function of RsgI9 requires further investigation.

It should be further noted that the studies presented in this paper are based on *in vitro* protease assays. The cellular environments of *C. thermocellum* are markedly different and more complex than those within a protease assay. Therefore, the precise reactions occurring within the cell may still require verification through genetic and other *in vivo* experiments. Our findings regarding the specific recognition of RsgI N-terminal fragments by ClpXP provide valuable insights for future *in vivo* studies.

Our study confirms the critical role of the ClpXP protease complex in the signal transduction of multiple RsgIs, while also highlighting the potential unique features of RsgI2 and RsgI9 that warrant further investigation. The number of RsgI factors differs among cellulosome-producing bacteria, and studies on different RsgIs in *C. thermocellum* can provide a framework for understanding the mechanisms of cellulosome dynamic regulation in other bacteria. These studies on bacterial transmembrane signal transduction and regulatory mechanisms are crucial for understanding the fundamental molecular mechanisms underlying the physiology and metabolism of these cellulose-degrading bacteria, thereby promoting their engineering and application in bioenergy production.

## Supplementary material

Supplementary Tables and Figures

## Data Availability

All relevant data are included within the main article and its Supplementary File.

## Abbreviations

LB: lysogeny broth
NC: nitrocellulose
NTD: N-terminal intracellular domain
RIP: regulated intramembrane proteolysis
RsgI-NFs: N-terminal fragments of RsgI
SDS-PAGE: sodium dodecyl sulfate-polyacrylamide gel electrophoresis
