# Supplementary material for: The final proteolytic step in transmembrane signaling of multiple RsgI anti-σ factors in Clostridium thermocellum
Source: Biosci Rep. 2025 Apr 9;45(4):233–45. doi: 10.1042/BSR20253055 (PMC12203970; doi:10.1042/BSR20253055)
Supplement: Supplementary Tables and Figures [file BSR-45-04-BSR20253055-s001.pdf]

Supplemental Materials

Table S1.

Bacterial strains used in this study.

**Strains**	**Relevant characteristic**	**Sources**
E. coli		
Top10	For plasmid construction	Transgen Biotech
BL21(DE3)	For protein expression	Transgen Biotech
Rosetta (DE3)	For protein expression	Transgen Biotech
Rosetta (DE3)::*pET28a-SMT3-rsgI1-NF-VAA*	Rosetta (DE3) strain containing a pET28a-SMT3 derivative for RsgI1-NF-VAA expression	This work
Rosetta (DE3)::*pET28a-SMT3-rsgI1-NF-VAA2*	Rosetta (DE3) strain containing a pET28a-SMT3 derivative for RsgI1-NF-VAA2 expression	This work
Rosetta(DE3)::*pET28a-SMT3-rsgI2-NF-FAA*	Rosetta (DE3) strain containing a pET28a-SMT3 derivative for RsgI2-NF-FAA expression	This work
Rosetta (DE3)::*pET28a-SMT3-rsgI2-NF-FAAA*	Rosetta (DE3) strain containing a pET28a-SMT3 derivative for RsgI2-NF-FAAA expression	This work
Rosetta (DE3)::*pET28a-SMT3-rsgI2-NF-FAAAA*	Rosetta (DE3) strain containing a pET28a-SMT3 derivative for RsgI2-NF-FAAAA expression	This work
Rosetta (DE3)::*pET28a-SMT3-rsgI2-NF-FAAAAA*	Rosetta (DE3) strain containing a pET28a-SMT3 derivative for RsgI2-NF-FAAAAA expression	This work
Rosetta (DE3)::*pET28a-SMT3-rsgI3-NF-IAA*	Rosetta (DE3) strain containing a pET28a-SMT3 derivative for RsgI3-NF-IAA expression	This work
Rosetta (DE3)::*pET28a-SMT3-rsgI4-NF-SAA*	Rosetta (DE3) strain containing a pET28a-SMT3 derivative for RsgI4-NF-SAA expression	This work
Rosetta (DE3)::*pET28a-SMT3-rsgI5-NF-SVA*	Rosetta (DE3) strain containing a pET28a-SMT3 derivative for RsgI5-NF-SVA expression	This work
Rosetta (DE3)::*pET28a-SMT3-rsgI6-NF-VAA*	Rosetta (DE3) strain containing a pET28a-SMT3 derivative for RsgI6-NF-VAA expression	This work

Rosetta (DE3)::*pET28a-SMT3-rsgI6-NF-VAA2*	Rosetta (DE3) strain containing a pET28a-SMT3 derivative for RsgI6-NF-VAA2 expression	This work
Rosetta (DE3)::*pET28a-SMT3-rsgI6-NF(1-57aa)*	Rosetta (DE3) strain containing a pET28a-SMT3 derivative for RsgI6-NF-(1-57aa) expression	This work
Rosetta (DE3)::*pET28a-SMT3-rsgI6-NF(1-70aa)*	Rosetta (DE3) strain containing a pET28a-SMT3 derivative for RsgI6-NF-(1-70aa) expression	This work
Rosetta (DE3)::*pET28a-SMT3-rsgI7-NF-VAA*	Rosetta (DE3) strain containing a pET28a-SMT3 derivative for RsgI7-NF-VAA expression	This work
Rosetta (DE3)::*pET28a-SMT3-rsgI8-NF-IAA*	Rosetta (DE3) strain containing a pET28a-SMT3 derivative for RsgI8-NF-IAA expression	This work
Rosetta (DE3)::*pET28a-SMT3-rsgI8-NF-IAA2*	Rosetta (DE3) strain containing a pET28a-SMT3 derivative for RsgI8-NF-IAA2 expression	This work
Rosetta (DE3)::*pET28a-SMT3-rsgI9-NF-IAA*	Rosetta (DE3) strain containing a pET28a-SMT3 derivative for RsgI9-NF-IAA expression	This work
Rosetta (DE3)::*pET28a-SMT3- rsgI6N-rsgI2TM-FAA*	Rosetta (DE3) strain containing a pET28a-SMT3 derivative for RsgI6N-RsgI2TM-FAA expression	This work
Rosetta (DE3)::*pET28a-SMT3- rsgI6N-rsgI9TM-IAA*	Rosetta (DE3) strain containing a pET28a-SMT3 derivative for RsgI6N-RsgI9TM-IAA expression	This work
BL21(DE3)::*pET28a-clpX*	BL21(DE3) strain containing a pET28a derivative for ClpX expression	This work
BL21(DE3)::*pET28a-clpE*	BL21(DE3) strain containing a pET28a derivative for ClpE expression	This work
BL21(DE3)::*pET28a-clpC*	BL21(DE3) strain containing a pET28a derivative for ClpC expression	This work
BL21(DE3)::*pET30a-clpP*	BL21(DE3) strain containing a pET30a derivative for ClpP expression	This work
BL21(DE3)::*pET28a-clpA*	BL21(DE3) strain containing a pET28a derivative for ClpA expression	This work
Rosetta (DE3)::*pET28a -MecA*	Rosetta (DE3) strain containing a pET28a derivative for MecA expression	This work
Rosetta (DE3)::*pET28a-SMT3-SpoIIAB*	Rosetta (DE3) strain containing a pET28a-SMT3 derivative for SpoIIAB expression	This work

Table S2.

Primers used in this study.

Primers	Sequences (5' to 3')	Notes
RsgI1-NF-VAA-F	GAGAACAGATTGGTGGATCC ATGAACAGATTGGGAATAAT ATATGAA	To construct the RsgIs-NF expression plasmids in *E. coli*
RsgI1-NF-VAA-R	TGGTGGTGGTGGTGCTCGAG CTAGGCAGCAACATATAAAA ATC	
RsgI1-NF-VAA2-F	GAGAACAGATTGGTGGATCC ATGAACAGATTGGGAATAAT ATATGAA	
RsgI1-NF-VAA2-R	TGGTGGTGGTGGTGCTCGAG CTATGCCGCAACACTTGAAA	
RsgI2-NF-FAA-F	GAGAACAGATTGGTGGATCC ATGTCACATTACACGGGAAT	
RsgI2-NF-FAA-R	TGGTGGTGGTGGTGCTCGAG CTAAGCGGCAAACGC	
RsgI2-NF-FAAA-F	GAGAACAGATTGGTGGATCC ATGTCACATTACACGGGAAT	
RsgI2-NF-FAAA-R	TGGTGGTGGTGGTGCTCGAG CTAGGCAGCGGCAAAC	
RsgI2-NF-FAAAA-F	GAGAACAGATTGGTGGATCC ATGTCACATTACACGGGAAT	
RsgI2-NF-FAAAA-R	TGGTGGTGGTGGTGCTCGAG CTAAGCGGCAGCGGCA	
RsgI2-NF-FAAAAA-F	GAGAACAGATTGGTGGATCC ATGTCACATTACACGGGAAT	
RsgI2-NF-FAAAAA-R	TGGTGGTGGTGGTGCTCGAG CTATGCAGCGGCAGCG	
RsgI3-NF-IAA-F	GAGAACAGATTGGTGGATCC ATGGATAACATAGGAGTAATC	
RsgI3-NF-IAA-R	TGGTGGTGGTGGTGCTCGAG CTAGGCTGCAATGCCGGTC	
RsgI4-NF-SAA-F	GAGAACAGATTGGTGGATCC ATGAATCTTGGAGTGGTAATA AAAATAAAAAG	
RsgI4-NF-SAA-R	TGGTGGTGGTGGTGCTCGAG CTAAGCTGCAGAATATGCAA GA	
RsgI5-NF-SVA-F	GAGAACAGATTGGTGGATCC ATGAAACACAAAGGCATTGT ATTAAAGC	

RsgI5-NF-SVA-R	TGGTGGTGGTGGTGCTCGAG CTAGGCAACGCTTAAAGCC
RsgI6-NF-VAA-F	GAGAACAGATTGGTGGATCC ATTGTAGGAAAAGTTCTTGATA
RsgI6-NF-VAA-R	TGGTGGTGGTGGTGCTCGAG CTATGCAGCAACTGCCGC
RsgI6-NF-VAA2-F	GAGAACAGATTGGTGGATCC ATTGTAGGAAAAGTTCTTGATA
RsgI6-NF-VAA2-R	TGGTGGTGGTGGTGCTCGAG CTATACAAAGCATGCAGCAACTGCC
RsgI6-NF-(1-57aa)-F	GAGAACAGATTGGTGGATCC ATTGTAGGAAAAGTTCTTGATA
RsgI6-NF-(1-57aa)-R	TGGTGGTGGTGGTGCTCGAG CTACGGCAAATATCTGCGCAAA
RsgI6-NF-(1-70aa)-F	TGCTTTGTAATTGTGTTGTCTTAGCTCGAGCACCACCACCACCACCACTGAG
RsgI6-NF-(1-70aa)-R	CTAAGACAACACAATTACAAAGCATGCAGCAACTGCCGCAACCGGCAAATAT
RsgI7-NF-VAA-F	GAGAACAGATTGGTGGATCC ATGAGGGCAATGGTAGTTGATA
RsgI7-NF-VAA-R	TGGTGGTGGTGGTGCTCGAG CTAAGCTGCTGCCACAGATA
RsgI8-NF-IAA-F	GAGAACAGATTGGTGGATCC ATGACAAAACAAAAAGGTACTATTTTAA
RsgI8-NF-IAA-R	TGGTGGTGGTGGTGCTCGAG CTATGCGGCAATTCGGGAATA
RsgI8-NF-IAA2-F	GAGAACAGATTGGTGGATCC ATGACAAAACAAAAAGGTACTATTTTAA
RsgI8-NF-IAA2-R	TGGTGGTGGTGGTGCTCGAG CTATGCGGCGATCCTTGCGGCAATTCGGGAATA
RsgI9-NF-IAA-F	GAGAACAGATTGGTGGATCC ATGAAGATAACCGGAGTCAT

	AGTAA	
RsgI9-NF-IAA-R	TGGTGGTGGTGGTGCTCGAGCTAGGCCGCAATCGATGCTA	
RsgI6N-RsgI9TM-IAA-F	TTCTCCAGAATAGCATCGATTGCGGCCTAGCTCGAGCACCACCACCACCACCACTGA	
RsgI6N-RsgI9TM-IAA-R	CTAGGCCGCAATCGATGCTATTCTGGAGAATCTGCGCAAAGAATTTTTAGGCTT	
RsgI6N-RsgI2TM-FAA-F	TTTTCTGCGTTTGCCGCTTAGCTCGAGCACCACCACCACCACCACTGAGATC	
RsgI6N-RsgI2TM-FAA-R	CTAAGCGGCAAACGCAGAAAATCTGCGCAAAGAATTTTTAGGCTTGATTATA	
ClpP-F	AAGAAGGAGATATACATATGAGTTTGGTACCGATAGT	For constructing the Clp and adaptor plasmids for expression of proteins used in ClpP protease assay
ClpP-R	TGGTGGTGGTGGTGCTCGAGTTTTCTTCTTTCCATAACTTCG	
ClpX-F	TGCCGCGCGGCAGCCATATGATACCAAAGCCCAGTGAAAT	
ClpX-R	TGGTGGTGGTGGTGCTCGAGTCAATTTATTATTACAGTCGGTGG	
ClpE-F	TGCCGCGCGGCAGCCATATGATGATGTGCTCAATTTGCAAA	
ClpE-R	TGGTGGTGGTGGTGCTCGAGTCATTCAGCGGTTGTGTTAT	
ClpC-F	TGCCGCGCGGCAGCCATATGTACGGACGTTTTACCGAAAAAG	
ClpC-R	TGGTGGTGGTGGTGCTCGAGTTAGCCCTTGTTTGAAACAAG	
ClpA-F	TGCCGCGCGGCAGCCATATGATGAGATTGGATGACGTAGC	
ClpA-R	TGGTGGTGGTGGTGCTCGAGTCACCTTTGAGTTTTTTTGCTTAT	
MecA-F	TGCCGCGCGGCAGCCATATGAAAATTGAAAAAATAAACGAAAATAAAATCAAAGTC	

MecA-R	TGGTGGTGGTGGTGCTCGAG TCATCTATGAAAATAGTCGTT TATTGTTTCCACC
SpoIIAB-F	GAGAACAGATTGGTGGATCC ATGAAAAATGAAATGCACCT TG
SpoIIAB-R	TGGTGGTGGTGGTGCTCGAG TTAATTACAAAGCGCTTTGC

Restriction sites are underlined and sequences of homology segments for seamless cloning are colored in red.

Table S3.

Plasmids used in this study.

Plasmids	Relevant characteristic
pET28a	The starting plasmid for constructing pET28a series plasmids
pET28a-SMT3	The starting plasmid for constructing pET28a-SMT3 series plasmids
pET28a-SMT3-RsgI1-NF-VAA	pET28a derivative for RsgI1-NF-VAA (residues1-59) expression in Rosetta (DE3) with N-terminal His tag and SMT3 tag
pET28a-SMT3-RsgI1-NF-VAA2	pET28a derivative for RsgI1-NF-VAA (residues1-66) expression in Rosetta (DE3) with N-terminal His tag and SMT3 tag
pET28a-SMT3-RsgI2-NF-FAA	pET28a derivative for RsgI2-NF-FAA (residues1-68) expression in Rosetta (DE3) with N-terminal His tag and SMT3 tag
pET28a-SMT3-RsgI2-NF-FAAA	pET28a derivative for RsgI2-NF-FAAA (residues1-69) expression in Rosetta (DE3) with N-terminal His tag and SMT3 tag
pET28a-SMT3-RsgI2-NF-FAAAA	pET28a derivative for RsgI2-NF-FAAAA (residues1-70) expression in Rosetta (DE3) with N-terminal His tag and SMT3 tag
pET28a-SMT3-RsgI2-NF-FAAAAA	pET28a derivative for RsgI2-NF-FAAAAA (residues1-71) expression in Rosetta (DE3) with N-terminal His tag and SMT3 tag
pET28a-SMT3-RsgI3-NF-IAA	pET28a derivative for RsgI3-NF-IAA (residues1-66) expression in Rosetta (DE3) with N-terminal His tag and SMT3 tag
pET28a-SMT3-RsgI4-NF-SAA	pET28a derivative for RsgI4-NF-SAA (residues1-60) expression in Rosetta (DE3) with N-terminal His tag and SMT3 tag
pET28a-SMT3-RsgI5-NF-SVA	pET28a derivative for RsgI5-NF-SVA (residues1-61) expression in Rosetta (DE3) with N-terminal His tag and SMT3 tag
pET28a-SMT3-RsgI6-NF-VAA	pET28a derivative for RsgI6-NF-VAA (residues1-60) expression in Rosetta (DE3) with N-terminal His tag and SMT3 tag
pET28a-SMT3-RsgI6-NF-VAA2	pET28a derivative for RsgI6-NF-VAAVAA (residues1-63) expression in Rosetta (DE3) with N-terminal His tag and SMT3 tag
pET28a-SMT3-RsgI6-NF-(1-57aa)	pET28a derivative for RsgI6-NF-(1-57aa) (residues1-57) expression in Rosetta (DE3) with N-terminal His tag and SMT3 tag

pET28a-SMT3-RsgI6-NF-(1-70aa)	pET28a derivative for RsgI6-NF-(1-70aa) (residues1-70) expression in Rosetta (DE3) with N-terminal His tag and SMT3 tag
pET28a-SMT3-RsgI7-NF-VAA	pET28a derivative for RsgI7-NF-VAA (residues1-59) expression in Rosetta (DE3) with N-terminal His tag and SMT3 tag
pET28a-SMT3-RsgI8-NF-IAA	pET28a derivative for RsgI8-NF-IAA (residues1-62) expression in Rosetta (DE3) with N-terminal His tag and SMT3 tag
pET28a-SMT3-RsgI8-NF-IAA2	pET28a derivative for RsgI8-NF-IAAGIAA (residues1-66) expression in Rosetta (DE3) with N-terminal His tag and SMT3 tag
pET28a-SMT3-RsgI9-NF-IAA	pET28a derivative for RsgI9-NF-IAA (residues1-158) expression in Rosetta (DE3) with N-terminal His tag and SMT3 tag
pET28a-SMT3-RsgI9-NF-IAAA	pET28a derivative for RsgI9-NF-IAAA (residues1-159) expression in Rosetta (DE3) with N-terminal His tag and SMT3 tag
pET28a-SMT3-RsgI6N-RsgI2TM-FAA	pET28a derivative for RsgI6N-RsgI2TM-FAA (residues63-68) expression in Rosetta (DE3) with N-terminal His tag and SMT3 tag
pET28a-SMT3-RsgI6N-RsgI9TM-IAA	pET28a derivative for RsgI6N-RsgI9TM-IAA (residues150-158) expression in Rosetta (DE3) with N-terminal His tag and SMT3 tag
pET28a-ClpX	pET28a derivative for ClpX (residues 61-409) expression in BL21(DE3) with N-terminal His tag
pET28a-ClpE	pET28a derivative for full-length ClpE expression in BL21(DE3) with N-terminal His tag
pET28a-ClpC	pET28a derivative for full-length ClpC expression in BL21(DE3) with N-terminal His tag
pET30a-ClpP	pET30a derivative for full-length ClpP expression in BL21(DE3) with C-terminal His tag
pET28a-ClpA	pET28a derivative for full-length ClpA expression in BL21(DE3) with N-terminal His tag
pET28a-ClpA-D2	pET28a derivative for ClpA-D2 (residues 466-776) expression in BL21(DE3) with N-terminal His tag
pET28a-MecA	pET28a derivative for full-length MecA expression in Rosetta (DE3) with N-terminal His tag
pET28a- SMT3-SpoIIAB	pET28a derivative for full-length SpoIIAB expression in Rosetta (DE3) with N-terminal His tag and SMT3 tag

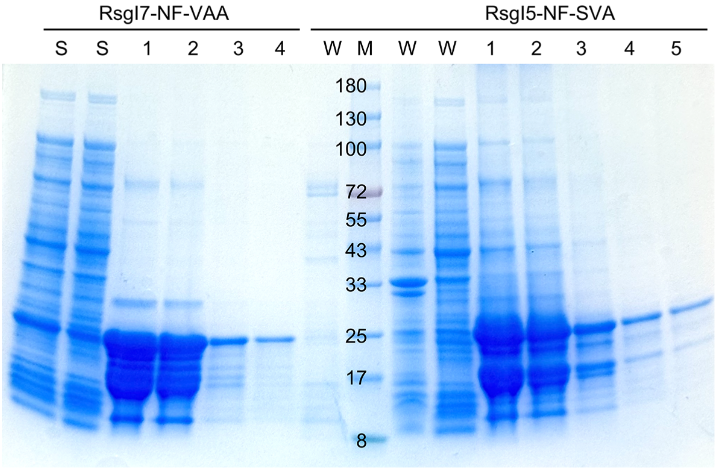

Fig. S1. Purification of RsgI5-NF-SVA and RsgI7-NF-VAA. The lanes 1-5 are the fractions eluted from the Ni^{2+} column at different times (from early to late). M, molecular weight marker; S, supernatant of the cell lysate; W, wash-out using a buffer that contains 30 mM imidazole.

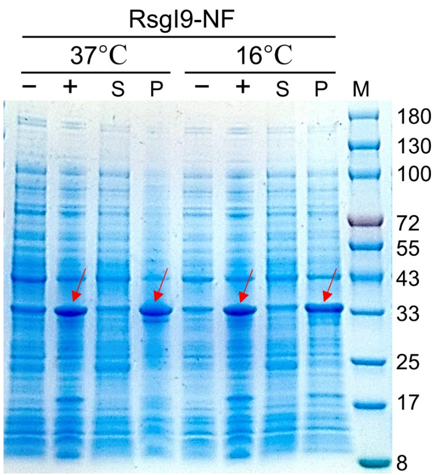

Fig. S2. Expression of RsgI9-NF-IAA at 37°C and 16°C. The red arrow indicates that the expression of RsgI9-NF-IAA protein is in the insoluble form. −: preinduction; +: postinduction; S: supernatant; P: pellet after centrifugation of the cell lysate.

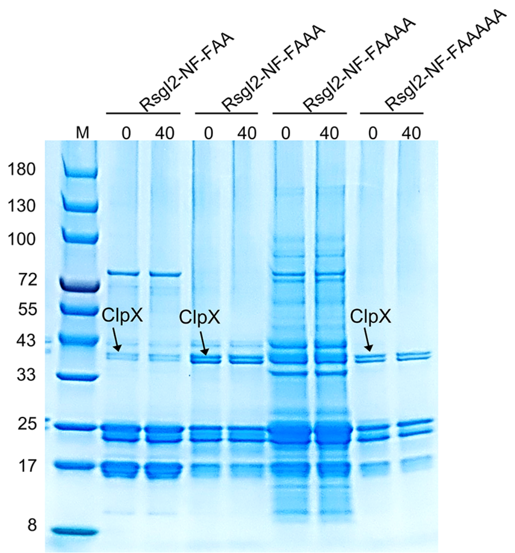

Fig. S3. ClpXP protease assay of RsgI2-NF-FAAA and RsgI2-NF-FAAAA. The bands of unfoldases are indicated by black arrows.

Fig.S4. Original uncropped pictures of the gels used in this study. The lanes and the corresponding figure which use them are indicated below the picture.

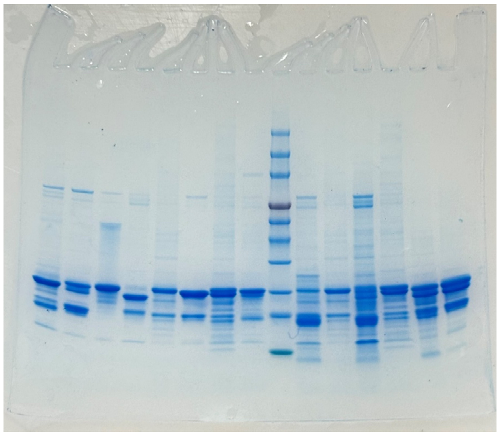

Lanes 1 to 9 are used to build **Fig.4. (A)**

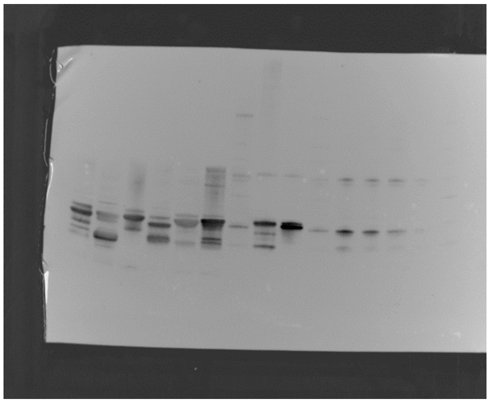

Lanes 1 to 9 are used to build **Fig.4. (B)**

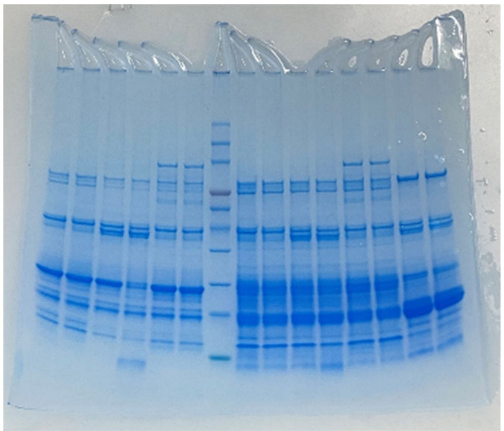

Lanes 1 to 7 are used to build **Fig.5. (A)**

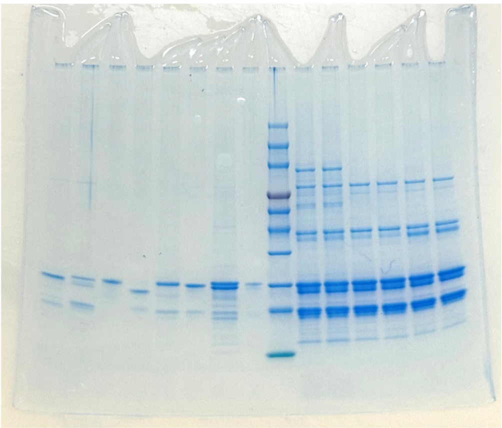

Lanes 9 to 15 are used to build **Fig.5. (B)**

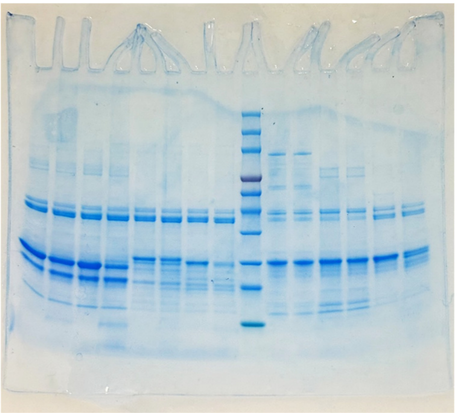

Lanes 9 to 15 are used to build **Fig.5. (C)**

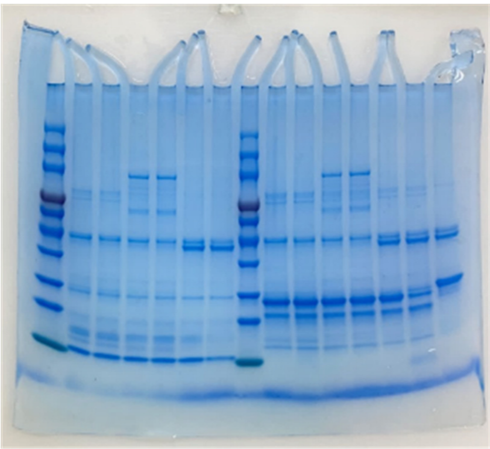

Lanes 8 to 14 are used to build **Fig.5. (D)**

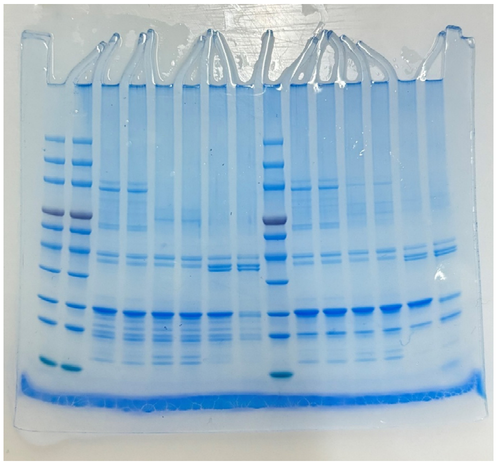

Lanes 9 to 15 are used to build **Fig.5. (E)**
Lanes 2 to 8 are used to build **Fig.5. (G)**

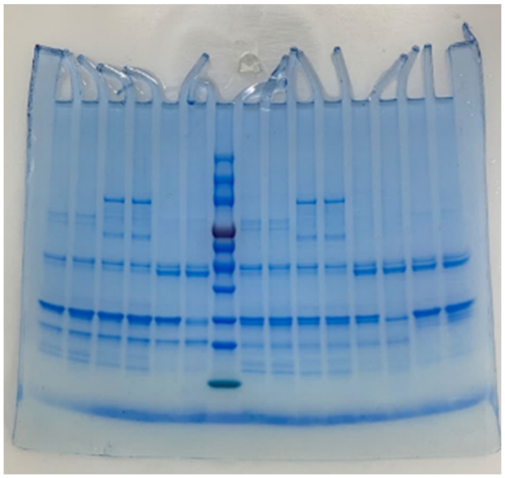

Lanes 1 to 7 are used to build **Fig.5. (F)**
Lanes 7 to 13 are used to build **Fig.5. (H)**

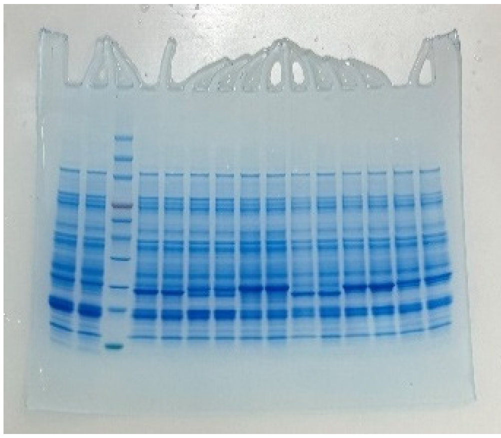

Lanes 1 to 15 are used to build **Fig.5. (I)**

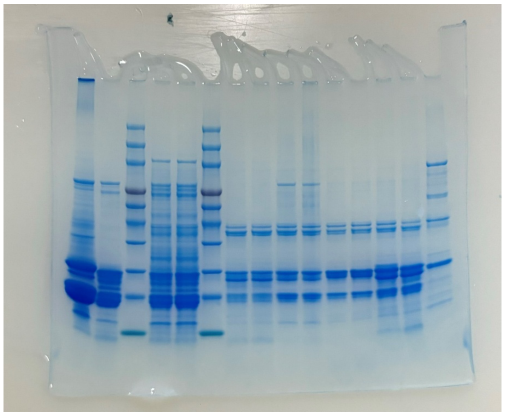

Lanes 3 to 5 are used to build **Fig.5. (I)**

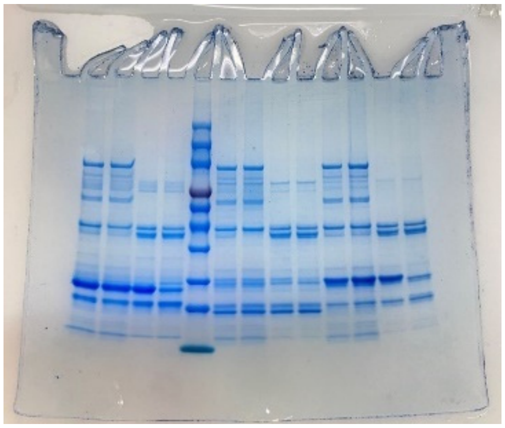

Lanes 1 to 5 are used to build **Fig.5. (J)**

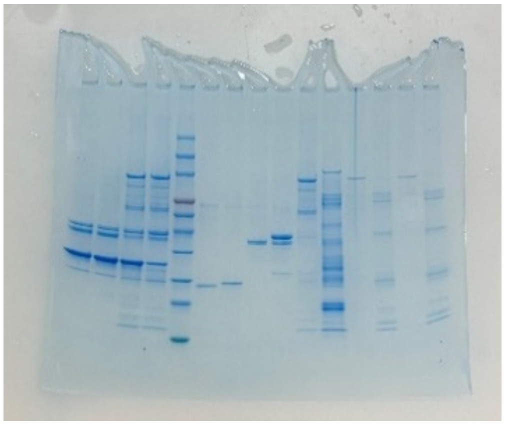

Lanes 1 to 5 are used to build **Fig.5(K)**

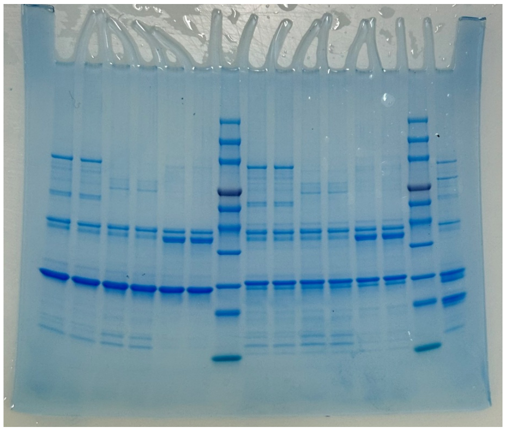

Lanes 1 to 14 are used to build **Fig.6.**

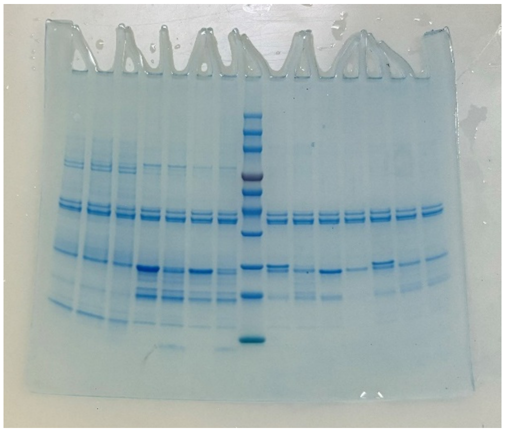

Lanes 4 to 12 are used to build **Fig.7. (A)**

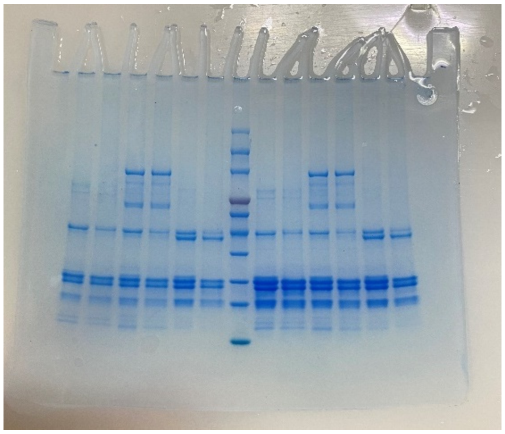

Lanes 7 to 13 are used to build **Fig.7. (B)**

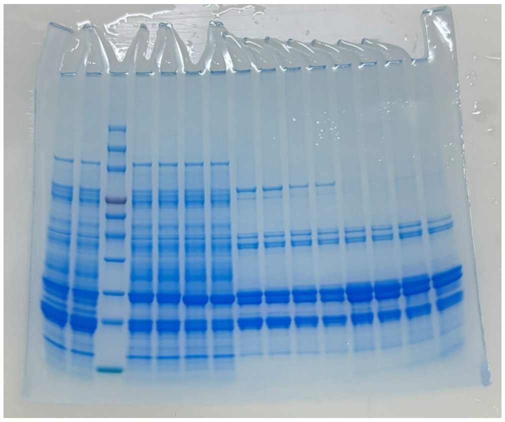

Lanes 3 to 7 are used to build the right panel of **Fig.8.**

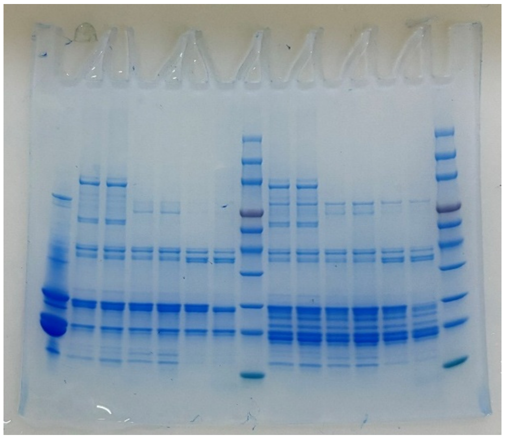

Lanes 2 to 8 are used to build the middle panel of **Fig.8.**

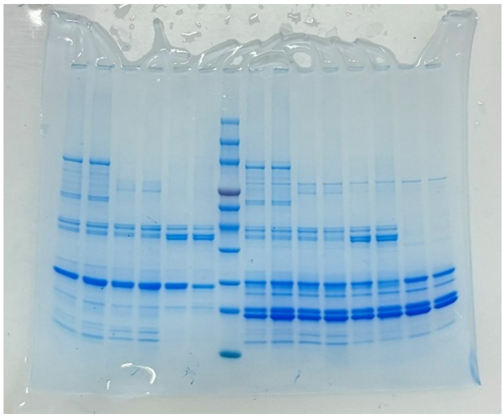

Lanes 1 to 7 are used to build the left panel of **Fig.8.**

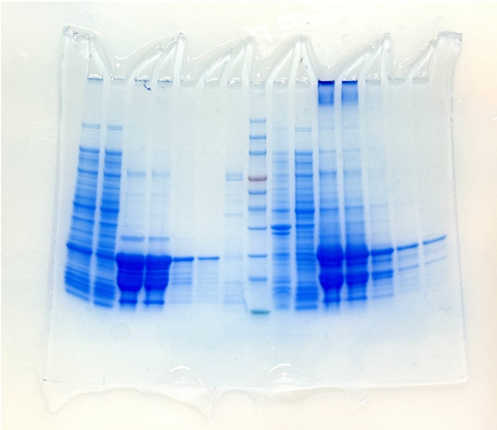

Lanes 3 to 11 are used to build **Fig.S1.**

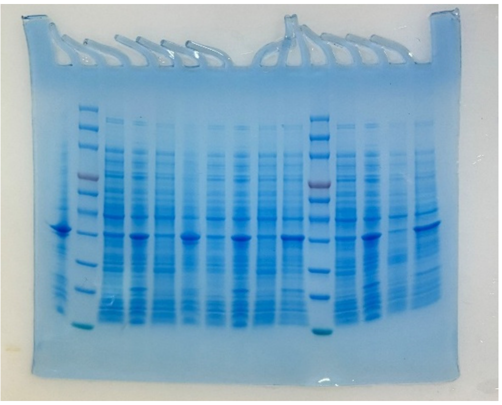

Lanes 3 to 11 are used to build **Fig.S2.**

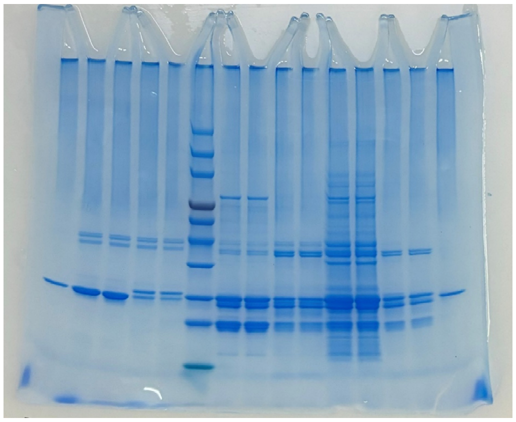

Lanes 6 to 14 are used to build **Fig.S3.**
